# Smoking and COPD Knowledge in the General Spanish Population: A CONOCEPOC Study

**DOI:** 10.3390/jcm12134473

**Published:** 2023-07-04

**Authors:** Juan Luis Rodríguez Hermosa, Marc Miravitlles, José Luis López-Campos, Myriam Calle Rubio

**Affiliations:** 1 Pulmonary Department, Research Institute of Hospital Clínico San Carlos (IdISSC), 28040 Madrid, Spain; jlrhermosa@yahoo.es; 2 Department of Medicine, Faculty of Medicine, University Complutense of Madrid, 28040 Madrid, Spain; 3 Pulmonary Department, Vall d’Hebron Research Institute (VHIR), Vall d’Hebron University Hospital, Vall d’Hebron Barcelona Hospital Campus, 08035 Barcelona, Spain; marcm@separ.es; 4 CIBER of Respiratory Diseases (CIBERES), Carlos III Health Institute, 28029 Madrid, Spain; lcampos@separ.es; 5 Medical-Surgical Unit for Respiratory Diseases, Institute of Biomedicine of Seville (IBiS), Virgen del Rocío University Hospital/University of Seville, 41013 Seville, Spain

**Keywords:** chronic obstructive pulmonary disease (COPD), tobacco use, knowledge, autonomous communities, rural and urban setting, respiratory symptoms, Spain

## Abstract

Background: The objective of this analysis is to evaluate tobacco use and the level of chronic obstructive pulmonary disease (COPD) knowledge among the general adult population in Spain and to compare these results to those obtained in the 2011 survey. Methods: A cross-sectional, observational, epidemiological study was conducted by telephone interviews and stratified by sex, age, and setting. The study design was identical to that of the study conducted in 2011. Results: Of a total of 89,601 phone contacts, there were 6534 respondents. The average age was 61.5 years. With respect to smoking, 30.9% reported being former smokers and 14.7% were current smokers, 63.6% of whom reported having attempted to quit. Among the current smokers, 19.7% claimed to use electronic cigarettes, although 88% believe these pose a health risk. No significant differences were found in smoking prevalence or frequency of attempts to quit according to residential setting (rural/urban). The highest prevalence of current smoking in men was recorded in the 55–64 years age range (31.6%), while in women it was from 45 to 54 years (34.6%). Smoking has decreased with respect to 2011, from 21.1% to 16.1% in men and from 17.9% to 13.2% in women, with a clear variability according to region. Of the population surveyed, 32.5% had spontaneous knowledge about COPD, with significant geographic variability. The most frequent sources of information about the disease were social media and the Internet (39.6%), followed by the media (35.2%). Conclusions: The prevalence of tobacco use in adults has considerably decreased and there is greater knowledge about COPD in Spain, although there is significant variability according to region, which could explain the geographic variability in the prevalence of COPD. Strategies are needed to increase COPD education and awareness and to reinforce smoking prevention measures among women.

## 1. Introduction

According to the World Health Organization (WHO) [1], tobacco use is the leading preventable cause of disease, disability, and premature death worldwide. In Spain, tobacco is the most commonly consumed psychoactive substance [2] and causes one million lost life years each year [3].

Since the 1950s, tobacco has been known to be the leading risk factor for developing COPD [4]. COPD is considered a health problem of primary importance due to its high prevalence and mortality, along with its elevated socio-health impact. Global mortality estimates indicate that COPD is the third leading cause of death [5,6], with 28,766 deaths per year in Spain due to the disease [7]. It is also responsible for elevated resource consumption, reaching three billion euros a year [8].

In Spain, two COPD prevalence studies carried out 10 years apart, EPISCAN I and II, determined the prevalences that continue to be significant for the general Spanish population between the ages of 40 and 80, 10.2% [9] and 11.8% [10], respectively, as well as the high variability between regions (2.4 times). Furthermore, the underdiagnosis of this disease has worsened by more than a point in ten years, reaching 74.7% of smokers and former smokers [10], despite actions undertaken over the years to improve disease diagnosis. The underdiagnosis of COPD may be related to a lack of knowledge about the disease.

In 2011, a study carried out in Spain showed that there was little knowledge about COPD and that many people with respiratory symptoms did not seek medical attention [11]. In the last 10 years, strategic interventions have been carried out by scientific societies and healthcare systems, such as information and awareness campaigns for the population, and recommendations to physicians to provide more active information on COPD and to increase the use of spirometry, together with the implementation of smoking cessation programs at the primary care level.

The objective of this study is to analyze tobacco use and the level of COPD knowledge among the general Spanish population aged 40–80 years. The secondary objectives are to compare these results to those observed almost ten years ago and, in turn, to increase awareness of the importance of tobacco use and COPD.

## 2. Materials and Methods

The methodology of the CONOCEPOC study has been extensively described elsewhere [12]. The development of the study follows the STROBE guidelines [13]. Briefly, CONOCEPOC is a cross-sectional, observational, epidemiological study promoted by the Spanish Society of Pulmonology and Thoracic Surgery (SEPAR), following a design identical to that of the study carried out in 2011 [11], with sampling obtained by randomly dialing landline phone numbers.

### 2.1. Subject Selection

The inclusion criteria were men and women over the age of 40 who agreed to answer a questionnaire over the phone. The survey was conducted over the phone in Spain’s 17 regions. Sampling was carried out in accordance with the following stratification criteria: sex, age according to decade (40–50, 51–60, 61–70, and >70 years), and rural (<10,000 inhabitants) or urban residential setting (≥10,000 inhabitants). Data, equally distributed by age, sex, and place of residence, were obtained in each of Spain’s 17 regions, requiring a total of 384 responses in each. This sample size allows identical precision of the samples by region in the population estimates, with an error of 5% and a power of 80% for the prevalences of the different variables higher than 5%, so that the overall sample obtained is representative of each of the regions and is stratified by age group and habitat (rural/urban).

### 2.2. Fieldwork

The phone interviews were conducted by Saatchi & Saatchi Health, Madrid, Spain, by qualified survey takers who underwent prior training. The call schedule was from 2 p.m. to 9:30 p.m. on weekdays, with an approximate duration of 15 min per interview. After randomly dialing a landline phone number in the corresponding geographic area, there were several options: if the number did not correspond to a home or residence, that number was randomly substituted; if no one answered the phone after a maximum of four attempts, the number was considered not contacted; the final option was the potential subject declining to participate. Finally, if no one eligible was present during the call, the number was recorded and called again according to the day/time the subject was expected to be at home.

### 2.3. Data Collection

Participants were asked about their spontaneous knowledge of COPD, without any guidance from the interviewer, by asking the question: “Do you know what COPD or chronic obstructive pulmonary disease?”. Those who spontaneously knew about COPD were asked to list the symptoms that are directly related to the disease. “Suggested” COPD knowledge was studied for the respondents who did not have spontaneous knowledge about the disease. The following sentence was read to them by the interviewer: As you know, COPD is the name for chronic obstructive pulmonary disease, which encompasses a group of diseases like chronic bronchitis and emphysema, and which is characterized by a feeling of shortness of breath, cough, wheezing while breathing and fatigue resulting from smoking and other causes. Does it sound familiar now? The measurement of tobacco use was evaluated by asking: do you smoke? How many cigarettes do you smoke a day? And how many years have you smoked? The survey was conducted in Spanish. In the CONOCEPOC studies of 2011 and 2021, in order to simplify the telephone interview, and similar to the National Health Survey [2,14] conducted in Spain for the population, the subjective perception that a person has about their general health status is collected through a simple scale. Perceived health and disease severity were evaluated on a scale from 0 to 10 points, where a higher score meant better perceived health or greater severity. Additionally, questions about new forms of tobacco use were included. The complete questionnaire is attached as Supplementary file.

### 2.4. Statistical Analysis

In the descriptive analysis, qualitative variables are presented with their frequency distribution. Quantitative variables are summarized with their average and standard deviation (SD) and quantitative variables that show an asymmetrical distribution are summarized with the median and interquartile range (IQR). Comparisons were made between study groups (by sex, setting, and smoking history). The association between qualitative variables was evaluated using the chi-squared test or Fisher’s exact test in the case that more than 25% of the expected frequencies were less than 5. Student’s t test was used for continuous variables in cases of variable normality; otherwise, non-parametric tests were used. A significance level of 5% was accepted for all tests. Data processing and analysis were carried out using IBM SPSS Statistics v21 software (IBM Corporation, Armonk, NY, USA).

## 3. Results

**Figure 1:**
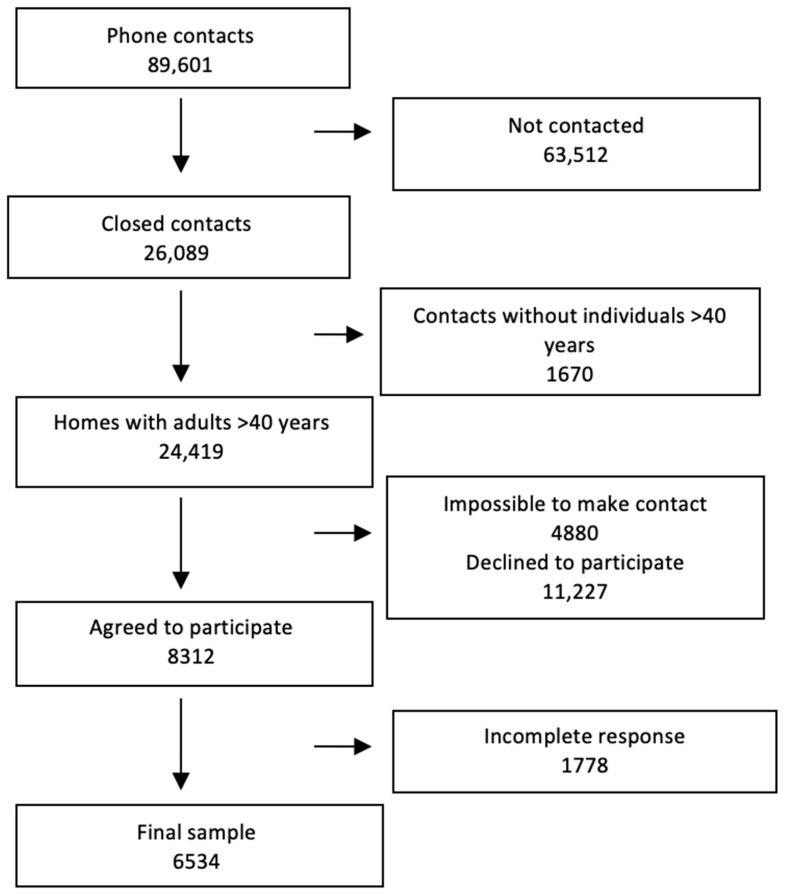
STROBE flowchart for the sample.

Of a total of 89,601 phone contacts, a final sample of 6534 responses was obtained, with a response rate of 26.7%, i.e., 6534 ultimate responses out of 24,419 homes identified with adults over the age of 40. The STROBE flowchart for participants and non-participants is presented in Figure 1.

### 3.1. Clinical Characteristics and Tobacco Use

A total of 50.4% of the respondents were women and the average age of the surveyed population was 61.5 years. With respect to tobacco, 54.4% had never smoked, while 14.7% were current smokers, with an average tobacco exposure of 19.3 pack-years. Among the current smokers, 63.6% had tried to quit the habit, with a median of two (1–3) attempts, and 4.5% reported having tried tobacco alternatives. A total of 24.8% reported some chronic respiratory symptoms, with an average perceived level of health of 7.4 (1.7). In Table 1, the characteristics of the subjects surveyed according to sex and residential setting are presented. The greatest prevalence of daily tobacco use among men (31.6%) was recorded in the 55–64 age range, while the highest prevalence in women was found in the 40–54 range (34.6%). The characteristics of the subjects surveyed in 2011 and 2021 are shown in Supplementary Table S1.

### 3.2. Evolution over Time of Tobacco Consumption

The overall prevalence of smoking in adults over the age of 40 in Spain for females versus males has gone from 17.9% versus 21.1% in 2011 to 13.2% versus 16.1% in 2021. The evolution of overall tobacco use prevalence figures over time, by region, and according to sex in 2021 and 2011 are shown in Table 2.

### 3.3. Characteristics according to Tobacco Use

The clinical and demographic characteristics of the individuals surveyed, according to their smoking history, are presented in Table 3. Compared to those who have never smoked, current smokers were younger (56.8 [10.7] vs. 62.4 [14] years, *p* < 0.05) and more frequently reported respiratory symptoms (40% vs. 20.7%, *p* < 0.001), although they less frequently reported having seen a doctor (42.3% vs. 55%, *p* < 0.001) or having gone to A&E (6.3% vs. 15.5%, *p* < 0.001) for respiratory symptoms.

### 3.4. COPD Knowledge

The overall frequency of spontaneous COPD knowledge at the national level increased, going from 17% in 2011 to 32.5% in 2021 (*p* < 0.05). The most frequent sources of information were social media and the Internet (39.6%), the media (35.2%), and family and acquaintances (19.5%). Perceived COPD severity was very high, with an average of 8.3 ± 1.5, only surpassed by angina pectoris. Statistically significant differences were observed for age, sex, residence area, smoking history, and respiratory symptoms according to the level of knowledge of COPD (Table 4). Compared to the survey conducted in 2011 [11], there were significant changes in spontaneous knowledge in all of the regions (Supplemental Figure S1), with significant geographic variability. Madrid (38.3%), La Rioja (38.8%), and Navarra (38.8%) were the communities with the highest levels of spontaneous COPD knowledge, while Catalonia had the lowest (19%) (*p* < 0.05).

## 4. Discussion

The main results of this analysis are that the prevalence of tobacco use in adults has decreased and there is more knowledge about COPD in Spain, although this change could be considered limited and varies significantly according to regions.

In our population of adults over the age of 40, the national average prevalence of daily tobacco use has decreased considerably with respect to 2011, dropping from 21.1% to 16.1% in men, and from 17.9% to 13.2% in women. These data reflect the gradual reduction shown in the recent National Health Surveys in Spain [2], with the prevalence of daily smoking in the population aged 15–64 going from 34% in 2017 to 32.3% in 2019, independent of sex or age. According to the 2020 Eurostat data14, Spain is among the countries with the most smokers in the European Union, with 19.7% of the population aged 15 and over smoking every day. Among the countries with the fewest daily smokers are Sweden (6.4%), Finland (9.9%), Luxembourg (10.5%), Portugal (11.5%), Denmark (11.7%), Ireland (13.8%), and the Netherlands and Belgium, with 14.6% in both cases. The average for the 27 countries that make up the European Union is 18.4%, which means that Spain is more than one percentage point higher.

In the analysis by sex, the greatest smoking prevalence in our population was recorded in men in the 55–64 age range (31.6%), while the highest prevalence in women was found in the 45–54 age range (34.6%). According to data from the latest European Health Survey conducted in 2020 [14], in Spain, the 25–34 age range shows the highest smoking prevalence in men (30.9%), while the highest prevalence in women is found in the 45–54 age range (23.8%). The prevalence in women in this age range is somewhat lower than in our results. This could indicate a rising trend in women that is worth monitoring and which could perhaps be explained by a certain bias in the implementation of smoking prevention measures, which are primarily focused on the primary care setting and a target population with cardiovascular risk that is predominantly male. These results should encourage the reinforcement of specific smoking cessation plans independent of programs involved in the prevention of cardiovascular risk, and with a greater intensity aimed at women.

Smoking is a complex addiction with socioeconomic determinants, such as socioeconomic status (level of schooling, occupation, employment, and income), and psychosocial aspects, such as social isolation, which have been proven to be factors associated with starting to use tobacco and influencing tobacco use cessation [15]. However, in our study, no significant differences were found in smoking prevalence or the frequency of attempts to quit according to residential setting (rural/urban).

In our population of daily tobacco users, 63.6% had considered quitting, with no differences according to sex or residential setting. In the latest official data evaluated in 2019 and collected in the last National Health Survey [2], 67.2% of respondents were reported to have considered quitting smoking, among daily tobacco users in the 35–64 age range, with a higher percentage among women (68.7%). The most effective measure to prevent COPD is tobacco cessation. However, although almost 70% of smokers want to quit, and half of those individuals try to quit at least once a year, only 1% are successful without any help, 3% manage to quit with only simple medical advice, 5–10% with minimal interventions, and 25–30% after intensive treatments including anti-tobacco drugs [15]. As a result, current guidelines recommend that all smokers who are in the process of quitting have access to anti-tobacco drugs [15,16]. Our study shows that there is a high number of active smokers who want to quit. A total of 63.6% of active smokers had tried to quit, with a median number of attempts of two (1–3). This provides a window of opportunity to act on, which should encourage administrations to be more active in reinforcing assistance programs in different fields of action to increase their accessibility and success.

With respect to electronic cigarette use, in our population, 4.5% (4.4% of men and 4.8% of women) have used them as an alternative to tobacco at least once in their lives, with no significant differences according to residential setting. This prevalence is far less than the 10.5% (12.0% of men and 8.9% of women) reported in the National Health Survey [2] for the population aged 15–64. This can be explained by the fact that the ages 15–24 group shows the highest prevalence of e-cigarette use. However, e-cigarette use prevalence is higher when analyzed in the daily tobacco users group. In our population, 19.7% of current smokers reported using e-cigarettes and 25% considered them a strategy to reduce tobacco use or to quit smoking, although a very high percentage believe they carry health risks. This prevalence is similar to the official one, where the prevalence of e-cigarette use is almost 21% among those who have smoked tobacco daily in the past 30 days [2]. The use of e-cigarettes has become a habitual smoking cessation method, seen in 32% of those who have tried to quit smoking [17].

Tobacco has been known to be the leading risk factor in developing COPD14. Its causal relationship has been established through numerous prospective cohort studies, including the Framingham Heart Study Offspring [18].

In our study, the prevalence of tobacco use and its evolution over time with respect to 2011, shows a significant variability by region. These differences in the prevalence and evolution of tobacco use according to region could be the main determinant of the variability in COPD prevalence in Spain found in the EPISCAN II [10] population study. In this study, disease prevalence was found to have a high variability (2.4 times) among the 17 regions, with the lowest prevalence of 7.1% in Asturias and the highest of 17.3% in Catalonia. In our analysis, the regions with the highest prevalence of COPD in men, such as Galicia (22.8%), Catalonia (22.7%), and Extremadura (21.1%), showed the lowest prevalence of subjects who had never smoked, with a slight increase in the number of men in this group of subjects in the past 10 years. On the contrary, in the region of Asturias, which has the lowest prevalence of COPD in men (9.1%) [10], the data from our survey show a significant increase in the population of men who have never smoked over the past 10 years (from 38.3% in 2011 to 51% in 2021).

However, other risk factors for this disease that have been described must be taken into account in addition to tobacco use, such as repeat lung infections during childhood or early adulthood, socioeconomic factors [19], and exposure to biomass or other combustibles used in heating or cooking in rural areas. In a study carried out in Galicia, up to 24% of patients with COPD had exposure to biomass smoke as an etiological factor [20]. This figure could explain the high prevalence of COPD in women in this community (10.2%) [10], despite the low prevalence of smoking in women (8.9%) and an increase in recent years in the population of women who have never smoked (from 64.7% in 2011 to 74.5% in 2021).

Also, other factors should be considered in this variability by region, such as air pollution. The association between long-term exposure to ambient air pollution and COPD incidence has been evaluated, and positive associations have been found, particularly for fine particulate matter < 2.5 mm in diameter, which could be an important modifiable risk factor for COPD [21].

In relation to the population’s level of knowledge about COPD, the results of our population study show that a lack of knowledge about COPD is still a reality for a large portion of the population. Only 32.5% of the Spanish population spontaneously know what COPD is, although this level of knowledge has almost doubled in the last 10 years (up from 17% in 2011) [11]. The impact of COPD on public health and its media presence are disproportionate. The main source of information on COPD is the Internet and social media, a channel that is growing considerably, because up to 55% of Spaniards use the Internet to search for health information [22]. The factors that determine a greater presence in the media are, on one hand, the release of news and relevant medical information related to a disease and, on the other, the fact that a disease is associated with well-known public figures. An additional disadvantage for COPD is its name, which is unfamiliar to large portions of the population. In our study, 555 of those interviewed had no spontaneous knowledge when referring to the acronym “COPD”, but did when referring to it by its full name, “chronic obstructive pulmonary disease”. A similar disadvantage has affected cerebrovascular accidents in recent years, where terms such as embolism or stroke are more well known among the population and have become everyday terms.

In our analysis, it must be highlighted that a clear variability can be seen according to region with respect to COPD knowledge, with less knowledge in Catalonia (19%), while Navarra (38.8%) and La Rioja (38.8%) are the regions with the most knowledge. Upon analyzing the changes that have taken place in the level of knowledge between 2011 [11] and 2021, a greater increase in knowledge is seen in Madrid, La Rioja, Navarra, and the Basque Country, autonomous communities that reported less underdiagnosis in the EPISCAN II study [10]. Less knowledge about COPD could serve as a determining factor in the underdiagnosis of a disease that is nevertheless considered severe by the subjects familiar with it. Seeing a doctor is still only reported by half of subjects with chronic respiratory symptoms [12,23].

This study used a methodology similar to that of the survey in 2011 [11], with sampling obtained by randomly dialing landline phone numbers. It is representative of the Spanish population since the objective was to analyze tobacco use and the level of COPD knowledge in the general adult population in Spain by region and to compare these results to those obtained in the 2011 survey. However, the results may be susceptible to bias, potentially leading to an inaccurate estimation of the true differences between the years. It was not adjusted for possible confounding factors, such as socioeconomic status, health literacy, and psychosocial aspects, which have been proven to be factors associated with the use of tobacco and medical knowledge. In addition, the methodology should be considered a limitation that could cause a bias in the population studied, since landline telephone use is declining in Spain due to the worsening of the economic crisis and only 77% of households in the country have a landline telephone, according to survey data from the National Institute of Statistics (INE) in 2019 [22]. However, it is important to note that response rates themselves are not a measure of survey quality. Researchers have demonstrated that telephone surveys with low response rates are still able to represent the entire population accurately [24]. Other limitations should also be considered, such as conducting the study during the period of the COVID-19 pandemic, as the pandemic has had a major impact on people’s mental and physical health, and this may have led to a change in smoking habits and health perceptions. Another factor to consider is health literacy, the ability of patients and their families to understand health and medical information. The European Health Literacy Survey (HLS-EU) showed that in Spain, 52% of the population had inadequate health literacy [25]. Low health literacy is related to health status and the relevant clinical outcomes of diseases because these people will have problems understanding health information [26]. It should also be kept in mind that this study evaluates self-reported responses without validation, although respondents provided honest estimations for tobacco use and level of COPD knowledge anonymously, in a large population that is representative of all autonomous communities, and with a response rate of 26.7%, higher than that of the previous survey in 2011 [11], which was 13.1%.

## 5. Conclusions

The prevalence of tobacco use in adults over the age of 40 in Spain has considerably decreased with respect to 2011, but this decrease is less among women and intensifying prevention measures in this group is therefore recommended. COPD knowledge among the Spanish population has nearly doubled in the past 10 years, although it is still lacking. There is considerable variability between regions, both in tobacco use and the level of COPD awareness, which may be determinants in the geographic variability of the prevalence and underdiagnosis of the disease. Strategies are needed to increase COPD education and awareness and the importance of maintaining smoking prevention measures and the reinforcement of smoking cessation among women in the primary care setting.

## Figures and Tables

**Table 1:** Characteristics of the population surveyed according to sex and urban/rural setting.

	Total	Women	Men	<10,000 Inhabitants	≥10,000 Inhabitants
N	6534	3296	3238	2758	3450
Sex (male), n (%)				1338 (48.5)	1787 (51.8) ^ß^
Age (years), (avg ± SD)	61.5 (13.1)	61.5 (12.9)	61.4 (13.2)	61.3 (13.3)	61.5 (12.8)
Smoking history					
Pack-years, (avg ± SD)	19.3 (18.7)	17.7 (19.7)	20.6 (17.7) *	17.7 (19.7)	20.6 (17.7)
Never smoked, n (%)	3554 (54.4)	2111 (64.1)	1443 (44.6) *	1549 (56.2)	1779 (51.6)
Former smoker, n (%)	2018 (30.9)	747 (27.2)	1271 (39.3) *	782 (28.4)	1179 (34.2)
Current smoker, n (%)	958 (14.7)	436 (13.2)	522 (16.1) *	427 (15.5)	492 (14.3)
From 40 to 44 years	123 (12.8%)	55 (12.6%)	68 (13%)	60 (14.1)	58 (11.8)
From 45 to 54 years	307 (32%)	151 (34.6%)	156 (29.9%)	141 (33)	153 (31.1)
From 55 to 64 years	310 (32.4%)	145 (33.3%)	165 (31.6%)	134 (31.4)	164 (33.3)
From 65 to 74 years	149 (15.6%)	71 (16.3%)	78 (14.9%)	61 (14.3)	82 (16.7)
From 75 to 84 years	64 (6.7%)	14 (3.2%)	50 (9.6%)	27 (6.3)	34 (6.9)
85 years and older	5 (0.5%)	0 (0%)	5 (1%)	4 (0.9)	1 (0.2)
Attempts to quit, n (%)	609 (63.6)	282 (64.7)	327 (62.6)	272 (63.7)	312 (63.4)
No. of attempts, median (P25–75)	2 (1–3)	2 (1–3)	2 (1–4)	2 (1–3)	2 (1–3)
Have you ever tried tobacco alternatives? (%)	4.5	4.4	4.8	29 (3.9)	55 (5.1)
Perceived level of health, (avg ± SD) ^¶^	7.4 (1.7)	7.4 (1.8)	7.3	7.34 (1.79)	7.48 (1.68)
Poor, n (%)	334 (5.1)	180 (5.5)	154 (4.8)	147 (5.3)	165 (4.8)
Average, n (%)	2537 (38.9)	1224 (37.2)	1313 (40.6)	1119 (40.6)	1287 (37.3)
Good, n (%)	3659 (56)	1890 (57.4)	1769 (54.7)	1492 (54.1)	1998 (57.9)
Some chronic respiratory symptoms, n (%)	1618 (24.8)	822 (24.9)	796 (24.5)	695 (25.2)	830 (24.1)
Have seen a doctor, n (%)	836 (51.6%)	420 (50.2)	416 (52.2)	334 (48.1)	446 (53.7)
Family medicine specialist	468 (56)	255 (60.7)	213 (51.2)	190 (56.9)	236 (52.9)
Pulmonologist	368 (44)	165 (39.3)	203 (48.8)	144 (43.1)	210 (47.1)

^¶^ Perceived health was evaluated on a scale from 0 to 10 points, with a higher score meaning better perceived health. * *p* < 0.01, men compared to women. ^ß^ *p* < 0.05, urban compared to rural.

**Table 2:** Smoking prevalence (%) by sex in 2021 and 2011 according to region.

	Smoker				Former Smoker				Never Smoked			
	Female		Male		Female		Male		Female		Male	
	2011	2021	2011	2021	2011	2021	2011	2021	2011	2021	2011	2021
Total	17.9	13.2	21.1	16.1	18.7	22.7	38.9	39.3	64	64.1	40	44.6
Andalusia	19.1	14.7	22.2	13.4	18.1	20.3	42.8	44.9	62.8	65.0	35	41.7
Aragon	19.6	11.5	18.3	19.8	20.1	23.4	47.1	41.7	60.3	65.1	40	38.5
Asturias	19.6	13.5	23.9	15.1	15.2	24.4	37.8	33.9	65.2	62.2	38.3	51.0
Canary Islands	14.2	12.6	21.7	18.2	13.2	20.4	32.8	26.6	72.5	67	45.6	55.2
Cantabria	22.1	14.6	15.6	15.6	20.6	23.4	42.8	33.3	57.4	60.9	41.7	52.1
Castilla-La Mancha	17.6	10.9	21.7	12.5	16.2	19.3	42.2	42.2	66.2	69.8	36.1	45.3
Castile and León	15.7	11.5	23.3	13.6	27	19.3	38.9	48.2	57.4	69.3	41.7	38.2
Catalonia	12.3	11.6	21.1	16.1	15.2	19.4	39.4	41.7	72.5	69.0	39.4	42.3
Madrid	19.6	14.6	23.9	13.0	18.6	27.1	38.3	49.5	61.8	58.3	37.8	37.5
Valencia	17.2	12.0	19.4	15.0	18.1	15.6	41.7	36.8	64.7	72.4	42.2	48.2
Extremadura	21.1	17.7	19.4	11.9	20.1	17.7	37.8	45.6	58.8	55.2	35.6	42.5
Galicia	18.6	8.9	18.9	17.7	16.7	16.7	38.9	40.1	64.7	74.5	42.2	42.2
Balearic Islands	19.6	16.6	23.3	14.1	14.2	19.7	36.1	37	66.2	63.7	40.6	49
La Rioja	17.6	13.5	18.3	18.2	17.2	29.2	41.1	37.5	65.2	57.3	40.6	44.3
Navarra	17.6	13.5	17.8	21.4	25	26.6	37.8	35.4	57.4	59.9	44.4	43.2
Basque Country	19.1	10.9	18.9	17.2	18.6	31.3	36.7	35.9	62.3	57.8	44.4	46.9
Murcia	14.2	15.6	23.9	22.4	13.7	22.9	35	38.0	72.1	61.5	41.1	39.6

**Table 3:** Clinical and demographic characteristics of subjects surveyed according to smoking history.

	Total (n = 6534)	Never Smoked (n = 3554)	Former Smoker (n = 2018)	Current Smoker (n = 958)
Sex (male), n (%)	3238 (49.5)	1443 (40.6)	1271 (63) *^¶^	522 (54.5) ^£^
Age (years), (avg ± SD)	61.5 (13.1)	62.4 (14.0)	62.2 (11.9) *^&^	56.8 (10.7) ^£^
Have tried to quit smoking	-	-	-	609 (63.6)
Number of attempts (avg ± SD)				3.3 (4.2)
Have you tried other forms of smoking? (%)	4.5	0.6	5 *^&^	19.7 ^£^
Attitude towards alternatives to traditional tobacco, (%)				
Highly favorable	0.3	0.2	0.5	0.4
Favorable	6.0	5.2	5.9	9.4
Do not know	44.2	47.3	42.1	37.5
Unfavorable	37.4	34.3	39.8	44.5
Highly unfavorable	11.8	12.9	11.7	8.2
Believe it can help quit smoking (%)	22.6	23.1	20.4	25.8 ^µ^
Believe it carries health risks (%)	88.5	87.7	89.9	88.6
Have chronic respiratory symptoms, n (%)	1618 (24.8)	736 (20.7)	499 (24.7)	383 (40) ^ß^
No. of respiratory symptoms, (%)				ß
0	75.2	79.3	75.3	60.0
1	17.2	15.1	18.5	22.4
2	4.5	3.5	3.6	10.4
3	2.5	1.3	2.0	4.9
4	0.9	0.8	0.6	2.2
Respiratory symptoms, (%)				
Chronic cough	17.1	18.6	14.9	18.3
Chronic expectoration	9.5	9.5	9.6	9.4
Whistling or chest sounds	12.9	12.9	11.8	15.8
Trouble breathing or shortness of breath	55.6	52.5	55.9	56.4
Feel short of breath on flat surface or at rest, n (%)	4.4	4.3	4.1	5.8
Have seen a doctor, n (%)	836 (51.6)	405 (55.0)	269 (53.9)	162 (42.3) ^ß^
Pulmonologist	468 (56)	176 (43.5)	132 (49.1)	60 (37) ^µ^
Family medicine specialist	368 (44)	229 (56.5)	137 (50.9)	102 (63)
Have gone to the emergency room for respiratory problems, n (%)	209 (12.9)	114 (15.5)	71 (14.2)	24 (6.3) ^ß^
Perceived level of health, (avg ± SD) ^Φ^	7.4 (1.7)	7.4 (1.7)	7.3 (1.6)	7.4 (1.8)

^Φ^ Perceived health was evaluated on a scale from 0 to 10 points, with a higher score meaning better perceived health. * *p* < 0.01, former smokers compared to those who have never smoked. ^¶^ *p* < 0.05, former smokers compared to current smokers. ^£^ *p* < 0.01, current smokers compared to those who have never smoked. ^µ^ *p* < 0.05, between groups, ^&^ *p* < 0.01 former smokers compared to current smokers; ^ß^ *p* < 0.01 between groups.

**Table 4:** COPD knowledge.

	2011 (n = 6528)	2021 (n = 6534)
Spontaneous knowledge about COPD, n (%)	1110 (17.0)	2120 (32.5)
Know symptoms of COPD, n (%)		
Trouble breathing	81.1	79.5
Cough	29	17.1
Expectoration	10.6	9.5
Whistling	17.2	13
Suggested knowledge about COPD, n (%)	2738 (41.9)	1696 (46.2)
Source of COPD knowledge, (%)		
Media	39.8	35.2
Social media or Internet	-	39.6
Doctor	23.6	5.2
Pharmacist	1.7	0.4
Relative/ acquaintance with disease	31.3	19.5
Perceived COPD severity, avg ± SD *	8.3 (1.5)	8.3 (1.5)
Perceived severity, avg ± SD		
Diabetes	7.6 (1.5)	7.7 (1.6))
Hypertension	7.5 (1.5)	7.5 (1.5)
Angina pectoris	8.8 (1.2)	8.7 (1.3)
Stomach ulcer	6.9 (1.5)	6.9 (1.6)
Arthrosis–arthritis	7.5 (1.5)	7.4 (1.6)

Data are expressed as the mean (standard deviation) or in absolute (relative) frequencies according to the nature of the variable. * Perceived disease severity was evaluated on a scale from 0 to 10 points, with a higher score meaning greater severity. Perceived level of health was evaluated on a scale from 0 to 10 points, where a higher score meant better perceived health.

## Data Availability

The datasets used and/or analyzed during the current study are available from the corresponding author on reasonable request.

## Supplementary Materials

The following supporting information can be downloaded at: https://www.mdpi.com/article/10.3390/jcm12134473/s1, Figure S1: Prevalence of spontaneous knowledge of COPD (%) by autonomous community in 2011 and 2021; Table S1: Characteristics of subjects surveyed.
