# Supplementary material for: Smoking and COPD Knowledge in the General Spanish Population: A CONOCEPOC Study
Source: J Clin Med. 2023 Jul 4;12(13):4473. doi: 10.3390/jcm12134473 (PMC10342498; doi:10.3390/jcm12134473)
Supplement: Supplementary file 1 [file jcm-12-04473-s001.zip › Appendix A.pdf]

**QUESTIONNAIRE**

*Hello, I’m ………………….*

*We’re conducting a study sponsored by the Spanish Pulmonology Society to research spirometry awareness. Participation in this study is voluntary and all data collected will be anonymous. The only drawback for you would be taking the time to answer a few short questions, which won't take more than 10 minutes.*

*Do you agree to participate and consent to your answers being recorded in order to analyze them anonymously?*

*Thank you very much.*

<u>SOCIODEMOGRAPHIC DATA</u>

S1 What is your age? _________

- < 40 ☐ years old→ ***End interview.***
- ☐ 40-50 years old
- ☐ 51-60 years old
- ☐ 61-70 years old
- >70 ☐ years old

S2.- Sex *(make note without asking based on name, if possible)*:

- ☐ Male
- ☐ Female

S3.- Autonomous community*:

- ☐ Andalusia
- ☐ Asturias
- ☐ Canary Islands
- ☐ Castilla-La Mancha
- ☐ Catalonia
- ☐ Galicia
- ☐ Aragon
- ☐ Madrid
- ☐ Cantabria
- ☐ Castilla y León
- ☐ Extremadura
- ☐ Balearic Islands

* This information is available in the phone number database for the general population.

- ☐ La Rioja
- ☐ Basque Country
- ☐ Valencia
- ☐ Navarra
- ☐ Murcia

S4.- Setting*:

- ☐ Up to 10,000 inhabitants
- ☐ over 10,000 inhabitants

<u>COPD AWARENESS - DIAGNOSIS</u>

C1.- On a scale from 0 (very poor) to 10 (very good), please indicate what you consider your general health status to be. └┴┘

C2.- Do you currently suffer from a respiratory disease?

- ☐ Yes → *Go to C3.*
- ☐ No → *Go to C4.*
- ☐ Don't know / No answer→ *Go to C4.*

**Spontaneous knowledge**

C3.- Please indicate which disease(s). *(**Don't** read options)*

- ☐ COPD (or chronic obstructive pulmonary disease)
- ☐ Asthma
- ☐ Chronic bronchitis
- ☐ Emphysema
- ☐ Other (indicate which other respiratory disease)

*If they **don't** indicate COPD, chronic bronchitis or emphysema in C3, answer C4.*

*If they indicate COPD in C3, go to C5.*

C4.- Do you know what COPD (chronic obstructive pulmonary disease) is? (record spontaneous knowledge)

- ☐ Yes What are the main symptoms? (don't read)

□ Morning cough□ Wheezing while breathing

□□Expectoration/Sputum/Phlegm □ Shortness of breath □ Other

If they answer YES for C4 (go to C6)

No (go to C5)

*C5. (Read) As you know, COPD is the name for chronic obstructive pulmonary disease, which encompasses a group of diseases like chronic bronchitis and emphysema, and which is characterized by a feeling of shortness of breath, cough, wheezing while breathing and fatigue resulting from smoking and other causes.*

Does it sound familiar now? □ No (go to C7) □ Yes (go to C6)

C6.- How did you learn about it?

□ Media (newspaper, radio, TV)

□ Internet or social media

□ Doctors

□ Pharmacist

□ Relative or acquaintance with disease

*C7.- Have you ever been diagnosed with the following diseases? Ask **only** about those **not** mentioned in C3.*

| | Yes | No | Don't know |
|---|---|---|---|
| COPD | □ | □ | □ |
| Asthma | □ | □ | □ |
| Chronic bronchitis | □ | □ | □ |
| Emphysema | □ | □ | □ |

*If COPD, chronic bronchitis or emphysema is **not** mentioned in C7 or in C2, go to E1.*

COPD TREATMENT *(Only if **COPD or chronic bronchitis or emphysema** is mentioned in C2 or C7)*

T1.- Do you follow a treatment plan for COPD?

□ No

□ Yes →T2. Please specify treatment (*read options*):

- Inhalers
- Medication
- Oxygen
- Support treatment to stop smoking

*If inhalers are indicated in T2 (go to T3).*

T3. On a scale from 0 (not at all difficult) to 10 (very difficult), please indicate your opinion of your inhaled treatment compliance.

Subject CHARACTERISTICS (for all respondents)

P1.- Do you smoke?

□ Yes, smoker P.1.1.1.- How many cigarettes do you smoke a day? _________

P.1.1.2.- How many years have you smoked? _________

P.1.1.3.- Have you ever tried to quit smoking?

□ Yes → P1.1.4.- How many times? _____________

□ No

□ No, former smoker

P 1.2.1 How long ago did you stop smoking?

P.1.2.2.- How many cigarettes did you smoke a day? ________

P.1.2.3.- How many years did you smoke?

□ No, never smoked

P2 Have you ever tried alternatives to cigarettes?

□ Yes → Specify *(read options)*:

- E-cigarettes
- IQOS
- JUUL
- Other

□ No

P.3 Please indicate your attitude towards these alternatives compared to traditional cigarettes.

□Highly favorable

□Favorable

□Don't know

□Unfavorable

□Highly unfavorable

P.4 Do you think they can help to stop smoking? (in regard to these alternatives to traditional cigarettes)

□ YES

□ NO

□Don't know

P.5 Do you feel there are health risks?
(in regard to these alternatives to traditional cigarettes)

□ YES

□ NO

□Don't know

P6.- Have you had a more or less constant morning cough for more than three

months a year for at least 2 years?

□ Yes □ No

P7.- Have you had more or less constant expectoration (cough with sputum) for more than three months a year for at least 2 years?

□ Yes □ No

P8.- Do you have more or less constant wheezing or noises while breathing for more than three months a year?

□ Yes □ No

P9.- Do you feel short of breath (trouble taking air in more than exhaling it)? *(Read options)*

□ No

□ When climbing a hill or two floors

□ When climbing one floor

□ When walking on a flat surface

□ At rest

*If any of the following symptoms are indicated: Yes for P6, P7, P8, or* ***not*** *indicating No for P9 (go to P10 and to P11)*

P10.- Have you seen a doctor for these problems? *(if necessary, reference cough, cough with sputum and/or shortness of breath, according to answer)*

□ No

□ Yes (specify):

Who?

□ primary care doctor

□ lung specialist/pulmonologist

Have you ever undergone spirometry (blowing into a device)?

□ No □ Yes

P11.- Have you ever gone to the emergency room for worsening of these

respiratory problems? *(if necessary, reference cough, cough with sputum and/or shortness of breath, according to answer)*

□ No

□ Yes (specify how many times in the past year):

COPD PERCEPTION (for all respondents)

F1.- On a scale from 0 (not serious) to 10 (maximum severity), please indicate how serious you believe COPD to be. _________

F2.- On a scale from 0 (not serious) to 10 (maximum severity), please indicate how serious you believe the following illnesses to be:

Diabetes…………….

Hypertension……….

Angina pectoris…

Stomach ulcer

Arthrosis-arthritis………

**Thank you very much for your participation.**
