# Supplementary material for: Smoking and COPD Knowledge in the General Spanish Population: A CONOCEPOC Study
Source: J Clin Med. 2023 Jul 4;12(13):4473. doi: 10.3390/jcm12134473 (PMC10342498; doi:10.3390/jcm12134473)
Supplement: Supplementary file 1 [file jcm-12-04473-s001.zip › FIGURE 1 Supplementary .pdf]

Supplementary Figure 1: Prevalence of spontaneous knowledge of COPD (%) by autonomous community in 2011 and 2021.

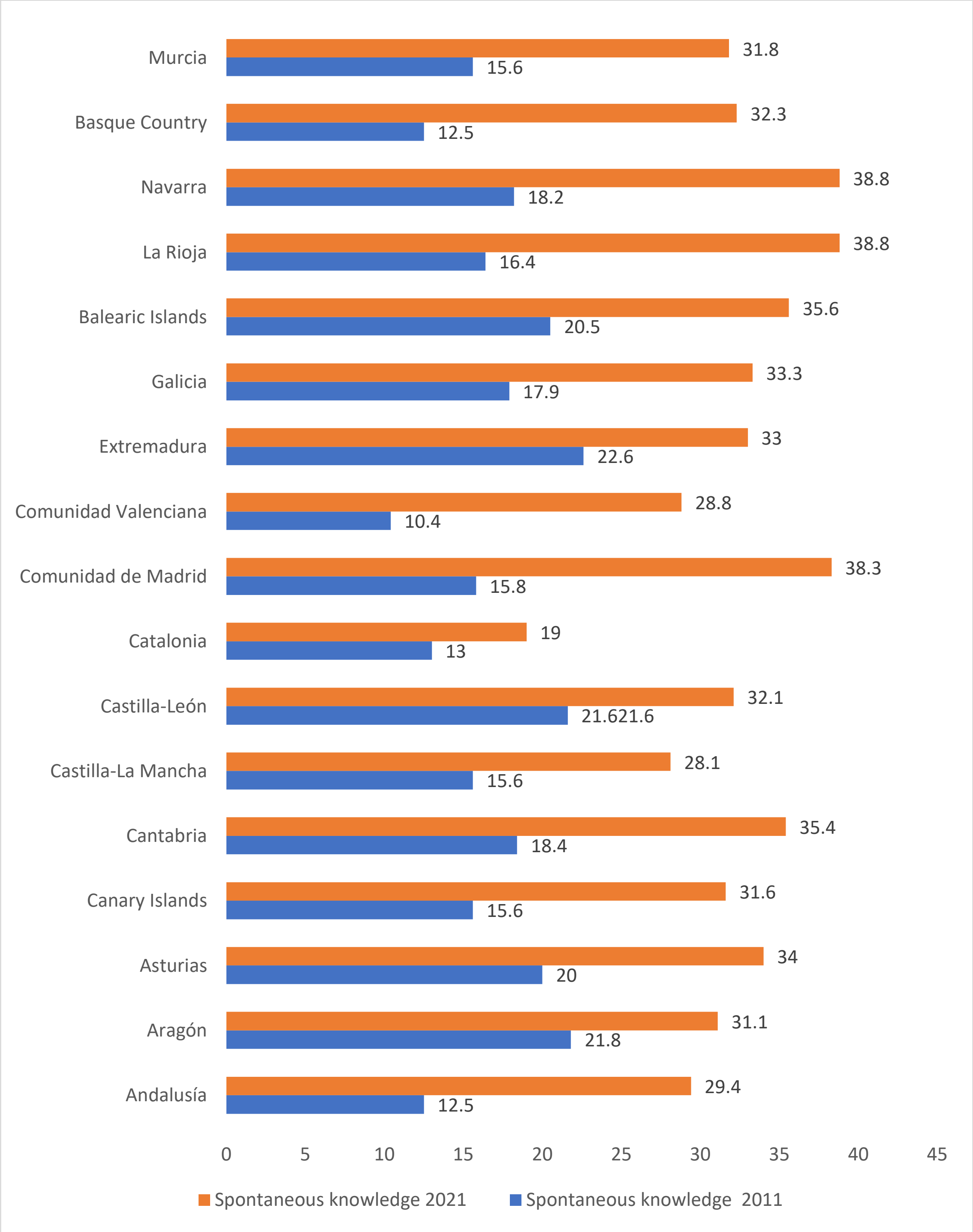

Data expressed in frequencies (%)
